# Pain in people living with HIV/AIDS: a systematic review

**DOI:** 10.7448/IAS.17.1.18719

**Published:** 2014-02-18

**Authors:** Romy Parker, Dan J Stein, Jennifer Jelsma

**Affiliations:** 1 Department of Health & Rehabilitation Sciences, Faculty of Health Sciences, University of Cape Town, Cape Town, South Africa; 2 Department of Psychiatry & Mental Health, Faculty of Health Sciences, University of Cape Town, Cape Town, South Africa

**Keywords:** prevalence, pain, HIV/AIDS, systematic review

## Abstract

**Introduction:**

Pain is one of the most commonly reported symptoms in people living with HIV/AIDS (PLWHA). However, wide ranges of pain prevalence have been reported, making it difficult to determine the relative impact of pain in PLWHA. A systematic review of the literature was conducted to establish the prevalence and characteristics of pain and to explore pain management in PLWHA.

**Methods:**

Studies that included cross-sectional data were included in the search, which was conducted in April 2012. Databases searched using a time limit of March 1982 to March 2012 included PubMed, Scopus, Africa-wide: NIPAD, CINAHL, PsychARTICLES, PSYCINFO, PSYCHIATRYONLINE, ScienceDirect and Web of Science. Search terms selected were “pain” and “HIV” or “acquired immune deficiency syndrome.” Two reviewers independently screened all citation abstracts for inclusion. Methodological quality was evaluated using a standardized 11-item critical appraisal tool.

**Results:**

After full text review, 61 studies fulfilled the inclusion criteria. Prevalence of pain ranged from a point prevalence of 54% (95%CI 51.14–56.09) to 83% (95%CI 76–88) using a three-month recall period. The reported pain was of moderate-to-severe intensity, and pain was reported in one to two and a half different anatomical sites. Moderate levels of pain interference with function were reported. All nine studies reporting on the adequacy of pain management recorded marked under-treatment of pain.

**Discussion:**

The studies reviewed reported that pain commonly presents at multiple pain sites with a range of severity suggesting that there are several differing pathological processes contributing to pain at one time. The interplay of variables associated with pain suggests that the biopsychosocial model of pain is an appropriate paradigm from which to view pain in PLWHA and from which to approach the problem, explore causes and establish effective treatment.

**Conclusions:**

The results highlight that pain is common in PLWHA at all stages of the disease. The prevalence rates for pain in PLWHA do not appear to have diminished over the 30 years spanning the studies reviewed. The body of work available in the literature thus far, while emphasizing the problem of pain, has not had an impact on its management.

## Introduction

Pain is a common symptom in people living with HIV/AIDS (PLWHA) [1]. The types of pain experienced by people with HIV/AIDS and the aetiology of such pains appear to vary. PLWHA may experience pain as a direct result of the virus on the peripheral or central nervous systems; pain may be due to immune suppression and resultant opportunistic infections; or pain may arise as a result of the side effects of anti-retroviral treatment (ART). The pain may also be idiopathic in origin with no clear aetiology or be due to other illnesses not associated with HIV [2].

Wide ranges of pain prevalence have been reported with figures as low as 0% [3] to above 90% [4]. With the range of prevalence rates reported, it is difficult to evaluate the relative impact of pain in PLWHA. A lack of information about the prevalence and impact of pain may impair treatment. In 1994, the International Association for the Study of Pain (IASP) formed a Task Force on Pain and AIDS in recognition of the need to disseminate information on pain in AIDS with a focus on addressing the under-management of pain in PLWHA. In a 1996 review article written for the IASP, Breitbart and Passik [5] emphasized the prevalence of pain in PLWHA and its under-treatment. Since then, several other studies reporting the prevalence of pain in PLWHA have been published and it appears that pain is still a significant problem for PLWHA and remains under-treated. However, these studies have been conducted in restricted populations in terms of ethnicity and stage of disease limiting the generalizability of the information. To date, no systematic review on the prevalence of pain in PLWHA has been published, resulting in a lack of information on the exact prevalence of pain and the extent of its under-treatment.

A systematic review of the literature was conducted to establish (1) the prevalence of pain, (2) the characteristics of pain, (3) factors that may contribute to pain, and (4) pain management in PLWHA. Secondary objectives included determining whether there has been a change in the prevalence of pain over time, whether there are differences in pain prevalence according to gender and whether different measurement instruments influence prevalence rates obtained.

## Methods

### Criteria for selecting studies for review

Descriptive cross-sectional surveys or longitudinal studies that included baseline cross-sectional data were included in the review. Surveys were included if they included PLWHA at any stage of the illness including those receiving ART, inpatient and outpatient groups, and adult males and females. Studies reporting on the prevalence of pain, characteristics of pain, factors contributing to pain, effects and consequences of pain and treatments for pain in PLWHA were included. Case-control studies, case reports and letters were excluded. Studies conducted at pain clinics or which had only included patients with pain or sub-groups of painful conditions (e.g. persons suffering from painful peripheral neuropathy only) were excluded because they would introduce a selection bias with the majority of participants presenting with pain.

### Search methods

A Medical Subject Heading (MeSH) search was conducted in PubMed to investigate search terms and assist in forming a search strategy. Search terms selected were “pain” and “HIV” or “acquired immune deficiency syndrome.” Electronic databases were selected for inclusion based on the likelihood of containing relevant information. Databases searched were PubMed (which includes Medline), Scopus (which indexes Embase), Africa-wide: NIPAD, CINAHL, PsychARTICLES, PSYCINFO, PSYCHIATRYONLINE, ScienceDirect and Web of Science. EBSCOHost was used for simultaneous searching of Africa-wide: NIPAD; CINAHL; PsychARTICLES; and PSYCINFO.

A time limit of March 1982 to March 2012 was set. Limits on the searches were set to include journal articles in English, “human” and “adult.” The terms “qualitative research”; “child” or “children” or “adolescent” or “neonate”; “rat” or “rodent” or “mouse”; “random* control* trial” or “case study” or “case report” were excluded using the “NOT” Boolean search term.

### Instrumentation for screening for inclusion and appraisal

All the citations identified were imported into Endnote^®^ and duplicates were removed. The first author and a research assistant independently screened all citation abstracts for inclusion using a form developed according to the inclusion and exclusion criteria (Table 1) and inter-rater reliability was established. The full texts of citations identified by either the first author, or the research assistant, as possibly containing relevant information were obtained after duplicates were removed.

**Table 1:** Criteria for screening of abstracts

Category	Inclusion criteria	Exclusion criteria
Population	HIV +	Sub-groups of HIV+ or reporting on HIV+ with pain only
Study design	Cross-sectional or longitudinal studies, prevalence studies	Case reports, case studies
Outcome	Report on the presence of pain in the sample	Reporting a pain sub-grouping only

There is a need for the use of rigorous guidelines when conducting systematic reviews and meta-analysis. The STROBE guidelines for the reporting of observational studies were consulted in order to evaluate quality of the selected citations [6]. The STROBE guidelines did not include specific information relating to pain; thus, methodological quality critical appraisal tools used in systematic reviews of epidemiological studies of pain or painful conditions were reviewed and used to inform the development of a tool [7–9]. The tool examined three methodological components of prevalence studies identified in both the STROBE guidelines [6] and other systematic reviews of prevalence studies [7–10], with 11 specific criteria (Table 2). Each criterion was marked as being present, absent or not applicable. Scores for each study were then calculated as a percentage. It is recommended that good quality prevalence studies should score a minimum of 70% on methodological appraisal [7, 9, 10]. However, there is a risk of excluding relevant data by setting a minimum score for selection. Therefore, the method described by Louw *et al*. [8] was used to select studies of acceptable standard for inclusion in the review. This entailed calculating the mean score of the studies selected, studies with scores of greater than or equal to the mean were included in the review.

**Table 2:** The 11-item critical appraisal tool

A. Is the sample representative of the target population?
i. Appropriate target population (HIV+ or AIDS adults)
ii. Reasons for non-responders and or description of non- responders
iii. Response rates stated
iv. Description of the population including HIV/AIDS stage or provision of CD4+ count, age, and sex of the sample
B. Methodological quality of the study
i. Study design (a cross-sectional study designed to collect prevalence data)
ii. Standardized method of data collection used
iii. The use of validated measurement instruments
iv. Data collected directly from the participants
C. Definition of pain as a symptom
i. The term “pain” operationally defined
ii. Inclusion of specific information relating to the pain such as frequency, location, intensity, and character of the pain
iii. Prevalence recall periods stated

Data were extracted from the studies and entered into spread sheets to create a summary table, which included the following information: year of publication, country and setting, sample size, age and gender, disease profile of the sample, outcome measure employed, pain prevalence and recall period, severity of pain, pain interference and pain management index (PMI). Differences in prevalence rates reported for each of the recall periods were tested using the Kruskall-Wallis test. Weighted mean prevalence rates of pain were calculated using the data from each of the studies. The prevalence data were summarized, pooled for analysis and reported systematically.

## Results

**Figure 1:**
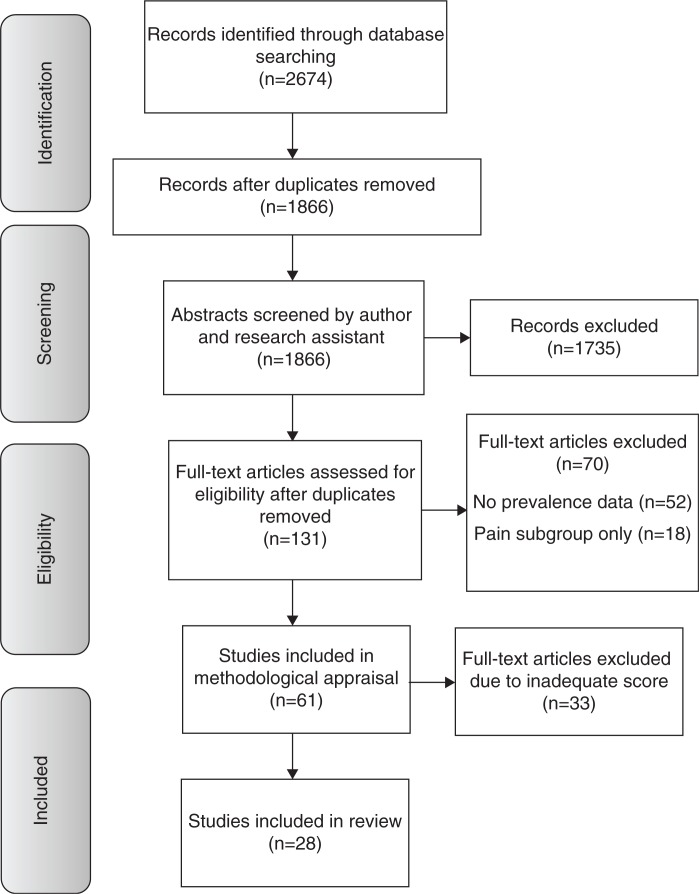
Flow diagram of the selection and review process.

The online search generated 2674 potential citations (Table 3). On removal of duplicates, 1866 citations remained. Following screening of the abstracts by two independent researchers using a previously piloted form developed according to the inclusion and exclusion criteria specified in Table 1, 134 citations remained and full text copies were obtained. Sixty-one studies fulfilled the inclusion criteria after full text review and were evaluated further using the 11-item critical appraisal tool. Following scoring with the critical appraisal tool, the mean score was calculated (68%) and the studies scoring greater than or equal to the mean were included in the review. The flow diagram of the selection and review process is presented in Figure 1. Summary scores of all 61 studies reviewed are presented in Supplementary file.

**Table 3:** Number of citations obtained from electronic databases

Database	Number of records identified
EBSCOHOST (Africa-Wide; CINAHL; Psycinfo; PsycARTICLES)	320
PubMed (which includes Medline)	141
PsychiatryOnline	38
ScienceDirect	240
Web of Science	1051
Scopus (which indexes EMBASE)	842
Total	2674

### Methodological appraisal of the studies

The 28 studies with scores greater than or equal to the mean score (68%) were selected for review. The minimum score on the appraisal tool for studies reviewed was 73% – above the 70% minimum recommended to indicate good quality prevalence studies [7, 9, 10]. Based on this appraisal, the studies accepted for this review were of high quality, strengthening the validity of the findings. A summary of the scores of the selected studies is presented in Table 4.

**Table 4:** Scores of selected studies using 11-item methodological appraisal tool

Author (year) reference	1. Entire target population/randomly selected sample/sample stated to represent target population	2. Non-responders	3. Response rate	4. Description of population	5. Primary data on the prevalence of pain in PLWHA?	6. The same mode of data collection	7. Validated questionnaire/interview/examination or at least tested for reproducibility or adequately described and standardized	8. Data collected directly from the participant?	9. Precise description of what is meant by pain?	10. Further useful specification on pain?	11. Prevalence recall periods stated	Score (%)
McCormack *et al*. (1993) [11]	1	0	0	1	1	1	1	1	1	1	1	82
Eldridge *et al*. (1994) [12]	1	1	0	1	1	1	1	0	0	1	1	73
Breitbart *et al*. (1996) [13]	0	1	1	1	1	1	1	1	1	1	1	91
Breitbart *et al*. (1997) [14]	0	0	0	1	1	1	1	1	1	1	1	73
Rosenfeld *et al*. (1996) [15]	0	1	1	1	1	1	1	1	1	1	1	91
Larue *et al*. (1997) [16]	1	1	0	1	1	1	1	1	1	1	1	91
Martin *et al*. (1999) [17]	1	1	1	1	1	1	0	1	1	1	0	82
Frich & Borgbjerg (2000) [18]	1	1	1	1	1	1	0	1	0	1	0	73
Rotheram-Borus (2000) [19]	1	1	1	1	0	1	0	1	0	1	1	73
Brechtl *et al*. (2001) [20]	1	1	1	1	0	1	1	1	1	0	1	82
Del Borgo *et al*. (2001) [21]	1	1	1	1	1	1	1	1	1	1	1	100
Cowdery & Pesa (2002) [22]	1	0	1	1	0	1	1	1	0	1	1	73
Lagana *et al*. (2002) [23]	1	1	1	1	0	1	1	1	1	0	1	82
Dobalian *et al*. (2004) [24]	1	1	1	1	1	1	1	1	0	1	1	91
Hughes *et al*. (2004) [25]	1	0	0	1	0	1	1	1	1	1	1	73
Aires & Bammann (2005) [26]	1	0	0	1	1	1	1	1	1	1	1	82
Jelsma *et al*. (2005) [27]	1	1	1	1	0	1	1	1	1	1	1	91
Hitchcock *et al*. (2008) [28]	1	1	1	1	1	1	1	1	1	1	1	100
Lee *et al*. (2009) [29]	1	1	0	1	0	1	1	1	0	1	1	73
Nair *et al*. (2009) [30]	1	0	0	1	1	1	1	1	1	1	1	82
Richardson *et al*. (2009) [31]	1	1	1	1	1	1	0	1	1	0	1	82
Aouizerat *et al*. (2010) [32]	1	1	0	1	1	1	1	1	0	1	1	82
Hansen *et al*. (2011) [33]	1	1	1	1	1	1	1	1	1	1	1	100
Miaskowski *et al*. (2011) [34]	1	1	1	1	1	1	1	1	1	1	1	100
Mphahlele *et al*. (2011) [35]	1	N/A	N/A	1	1	1	1	1	1	1	1	100
Narasimooloo *et al*. (2011) [36]	1	0	0	1	1	1	1	1	1	1	1	82
Tran *et al*. (2011) [37]	1	1	1	1	0	1	1	1	0	0	1	73
Wahab & Salami (2011) [38]	0	0	0	1	1	1	1	1	1	1	1	73

The prevalence rates in the studies not selected for review were compared with the rates of the studies selected for review. There was no significant difference between either the median or the mean prevalence rates (point prevalence, one-week, two-week and one-month prevalence rates) of the studies selected for review and those not selected. The most common weaknesses of the 33 studies excluded from the review were in the areas of reporting response rates and reporting the characteristics of non-responders. A lack of information in these criteria influences the validity of study findings because non-responders may have been in better health than responders and have reported no pain, resulting in a biased prevalence rate.

### Description of the studies

Descriptive data extracted from the 28 reviewed studies are grouped according to recall periods used and presented in chronological order in Table 5. Three of the studies reviewed reported on the same data set in New York [13–15], two studies reported on the same data set in Cape Town [25, 27] and a further two studies reported on the same data set in San Francisco [33, 34]. Data from the studies reporting on the same data sets were combined to prevent replication, resulting in 24 samples reporting on a total of 6814 PLWHA.

**Table 5:** Summary of reviewed studies

Author (year) reference	Setting	Population (sample size)	Age (years) (mean±SD or range)	Female (%)	Ethnicity (%)	Disease stage (%) and/or CD4+ count (cells/mm^3^) (mean±SD) or [median (range)] or CD4+<200 (%)	Time since diagnosis (months)	On HAART (%)	Measurement instrument	Pain prevalence (%) (period)
**Point prevalence**										
Eldridge *et al*. (1994) [12]	Boston, USA	Terminal AIDS patients in residential hospice (50)	37.6	25	Black (16) Hispanic (14) White (38) Other (4) Unknown (28)	AIDS	Missing	Missing	Interview	40 (point prevalence)
Frich & Borgbjerg (2000) [18]	Copenhagen, Denmark	AIDS patients at a Dept. of Infectious Diseases (95)	40±9	12.6	Danish (87) Scandinavian (4) African (5) Other (3)	AIDS	19.7±18.7 (pain) 14.8±13.9 (no pain)	50	Interview	74 (point prevalence)
Del Borgo *et al*. (2001) [21]	Rome, Italy	HIV+ admitted to ward or day treatment centre (153)	36±6.5	28.1	Missing	Groups A and B (23.5) Group C (76.5)	Missing	34	IPQ (Italian MPQ)	60.80 (point prevalence)
Hughes *et al*. (2004) [25]+	Khayelitsha, Cape Town, South Africa	HIV+ outpatients initiating HAART (123)	33.8±7.8	65	Missing	AIDS (100) CD4+<200 (100)	Missing	Naive	EQ-5D	69.10 (point prevalence)
Jelsma *et al*. (2005) [27]+	Khayelitsha, Cape Town, South Africa	HIV+ OP on HAART (117)	Missing	74.5	Missing	AIDS	Missing	100%	EQ-5D	54.9 (point prevalence at one month post-initiation of HAART) 46.4 (at three months) 39.8 (six months) 26.5 (one year)
Mphahlele *et al*. (2011) [35]‡	Urban and rural South Africa	HIV+ OP (rural 125; urban 396)	36±9 (rural) 36±8 (urban)	79 (rural) 75 (urban)	Black (100) (rural) Black (93) (urban)	[199 (120–346)] (rural) [200 (99–309)] (urban)	Missing	53 (rural) 68 (urban)	WBPI	72 (rural) 66 (urban) (point prevalence)
Narasimooloo *et al*. (2011) [36]	Kwazulu-Natal South Africa	HIV+ IP urban district hospital (100)	21–30 (34%) 31–40 (47%) 41–50 (16%) > 50years (3%)	66	Missing	Stage II (3) Stage III (29) Stage IV (68) CD4+<200 (70)	42% HIV+<six months	34	BPI	91 (point prevalence)
Tran *et al*. (2011) [37]	Vietnam	Nationally representative sample of adults LWHA (400)	30 (27–33)	37.3	Missing	Asymptomatic (43.8)	60 (95% CI 56.4–63.6)	56.25	EQ-5D	15.5 (point prevalence) [19.1 (on HAART) 10.9 (not on HAART)]
**One-week recall period**										
Larue *et al*. (1997) [16]	13 cities across France	HIV+ IP and OP (290)	33 (21–66)	22	Missing	Asymptomatic (3; 3; 20) Pathologic (5, 16, 23) AIDS (80; 75; 50) IP; DP; OP	Missing	Missing	BPI (French)	62; 53; 30 IP; DP; OP (one week)
Lee *et al*. (2009) [29]	San Francisco, USA,	Living with HIV/AIDS for minimum of three months, attending HIV clinics and community sites (317)	45.1±8.3; 45.9±8.2; 43.3±9.1 (M; F; Trans)	24.6	Black (28; 61; 61) Hispanic (11; 8; 13) White (50; 23; 13) Mixed (8; 4; 9) Other (3; 4; 4) (M; F; Trans)	AIDS 56; 46; 35 CD4+ (462±275); (439±246); (380±232) (M; F; Trans)	12.4±7.0; 11.1±6.5; 11.5±7.4 (M; F; Trans)	71	MSAS	55 (one week)
Nair *et al*. (2009) [30]	South India	IP and OP at HIV care centres, 90% ambulatory (42 IP, 98 OP)	18–68	41	Missing	Stage I (35); Stage II (11.5); Stage III (25); Stage IV (27)	Missing	60 (those with pain)	BPI	66.7 (IP) 24.5 (OP) (one week)
Aouizerat *et al*. (2010) [32]	San Francisco, USA	HIV+ OP (350)	45.3	24.6	Black (39) Hispanic (10) White (41) Other (10)	AIDS (52); (449±265)	12.1±7	71	MSAS	55 (one week)
Hansen *et al*. (2011) [33] and Miaskowski *et al*. (2011) [34]#	San Francisco, USA	Indigent PLWHA (296)	49.5±7.5 (29.3% F)	28.1	Black (41.9) White (38.1) Other (20)	HIV+ CD4+<200 (54.8)	Missing	74.8	BPI	91.2 (one week)
**Two-week recall period**										
Breitbart *et al*. (1996) [13] and Rosenfeld *et al*. (1996) [15]*	New York, USA	Ambulatory AIDS patients (438)	38.83	36.1	Black (37) Hispanic (22.9) White (38.1) Other (2.3)	AIDS [150 (0–1929)]	52.8	53.9	BPI	62.6 (two weeks)
Breitbart *et al*. (1997) [14]*	New York, USA	Ambulatory AIDS patients (516)	38.96±9.7(IDU) 38.13±7.9 (non-IDU)	49.5 (IDU) 39 (non-IDU)	Black (39.6; 39.4) Hispanic (27.4; 17.5) White (31.1; 39.8) Other (1.9; 3.3) (IDU; non-IDU)	AIDS CD4+<200 (31.1; 71.1) (IDU; non-IDU)	51.05±30.6 (IDU) 54.51±33.9 (non-IDU)	Missing	BPI	63.6 (two weeks) [67 (IDU); 59 (non-IDU)]
Brechtl *et al*. (2001) [20]	New York, USA	AIDS patients at discreet nursing unit (50)	34	33	Black (44) Hispanic (23) White (9) Unknown/mixed (24)	AIDS CD4+ [35(0–265)]	Missing	Naïve	BPI	62 (two weeks)
Aires & Bammann (2005) [26]	Sao Paulo, Brazil	HIV+ IP on admission (197)	34	26	Missing	HIV+	Missing	Missing	Modified WBPQ	54.3 (two weeks)
Hitchcock *et al*. (2008) [28]	Pretoria, South Africa	AIDS patients initiating HAART (354)	36.3±8.6	Missing	Missing	AIDS (111±70.8)	Missing	Naive	Missing	62.1 (two weeks)
Wahab & Salami (2011) [38]	Ilorin, Nigeria	PLWHA attending OP clinic of the University Hospital (79)	37.1±8.6	59.5	Missing	Stage I (43) Stage II (35.4) Stage III (17.7) Stage IV (3.8) (234.9±218.3)	28.5±25.3	Missing	Modified BPI	27.8 (two weeks)
**One-month recall period**										
Cowdery & Pesa (2002) [22]	South-eastern USA	Women receiving routine HIV treatment in a public clinic (82)	37.5±9.3	100	Black (67.1) White (32.9)	A (42.2) B (28.2) C (28.2) (402.4±363.6)	36.9±29.4	Missing	MOS-SF-20	63 (one month)
Dobalian *et al*. (2004) [24]	USA	HIV+ OP nationally representative sample (2267)	39	22.4	Black (32.2) Hispanic (15) White (49.3) Other (3.5)	AIDS (48.3) CD4+<200 (53.8)	Missing	Missing	SF-36	67 (one month)
Mphahlele *et al*. (2011) [35]‡	Urban and rural South Africa	HIV+ OP (rural 125; urban 396)	36±9 (rural) 36±8 (urban)	79 (rural) 75 (urban)	Black (100) (rural) Black (93) (urban)	[199 (120–346)] (rural) [200 (99–309)] (urban)	Missing	53 (rural) 68 (urban)	WBPI	67 (rural) 77 (urban) (one month)
**Three-month, six-month and unspecified recall periods**										
Martin *et al*. (1999) [17]	Stockholm, Sweden	HIV+ OP attending Dept. of Infectious Diseases (255)	F 38; M 40	22	Missing	Asymptomatic 53; Symptomatic 32; AIDS 15	Missing	Missing	Questionnaire	85; 71 IDU, non-IDU
Rotheram-Borus *et al*. (2000) [19]	New York, USA	Low-income advanced HIV or AIDS (151)	37.6±5.2	86.8	Black (39) Hispanic (41) White (9) Other (1)	32% AIDS CD4+<200 (61)	15±22 (AIDS diagnosis)	Missing	Missing	83 (three months)
Lagana *et al*. (2002) [23]	Northern California, USA	HIV+ OPs recruited for psychotherapy intervention study (120)	40±7.5	42.5	Black (31.7) Hispanic (10.8) White (57.5) Native American (7.5) Other (10)	Missing	74.7±42.5	75	Rating of chronic pain	48.3 (chronic pain>6 months)
Richardson *et al*. (2009) [31]	New York, Chicago, Washington DC, LA; USA	Women living with HIV (104+66+68+101=339)	62.8%<39	100	Black (53.7) Hispanic (29.5) White (16.5)	Asymptomatic (35) CD4+<200 (57.5)	Missing	60	Missing	83.5 (six months)

* Same sample

+ same sample

# same sample

‡ same sample OP=outpatients; IP=inpatients; M=male; F=female; Trans=transgender; IDU=intravenous drug user.

**Figure 2:**
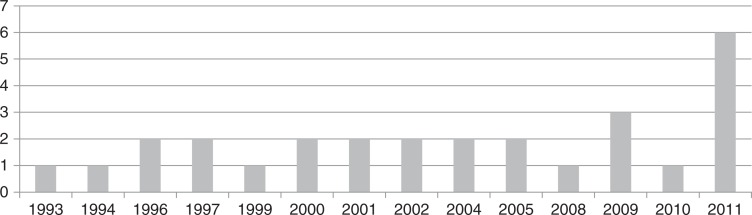
Number of studies included in the review per year.

None of the studies reviewed were published prior to 1993; 13 of the studies were published between 1993 and 2002; four studies were published between 2003 and 2007; and the remaining 11 studies were published between 2007 and 2011. In 2011 alone, six studies were published (Figure 2). The majority (19) of the studies were conducted in high-income countries with fourteen from the United States; one from Canada; one from France; one from Denmark; one from Sweden; and one from Italy. The remaining studies were conducted in the lower income countries of South Africa (five); Brazil (one); India (one); Vietnam (one); and Nigeria (one). The majority of the studies were conducted in urban settings with only one study reporting on a combination of rural and urban settings [35]. Sample sizes ranged from 50 participants in a single setting [12, 20] to nationally representative samples of 400 in Vietnam [37] and 2267 from the United States [24]. Only two of the studies reviewed included nationally representative samples of PLWHA [24, 37]. The remainder of the studies used samples of convenience recruiting participants from health care centres.

As per the inclusion criteria all the participants in the studies were either HIV+ or had been diagnosed with AIDS. Several mechanisms contributing to pain have been identified as differing between females and males with chronic pain conditions having a higher prevalence in females [39]. It is relevant therefore to explore gender distribution in the studies reviewed. In the studies reported on in this review, females made up 38% of the total sample. Females made up less than 50% of the sample in 18 of the studies. Less than 25% of the sample was female in seven studies [12, 16–18, 24, 29, 32] and between 25% and 50% of the sample was female in 11 of the studies [13–15, 20, 21, 23, 26, 30, 33, 34, 37].

### Prevalence and characteristics of pain

**Figure 3:**
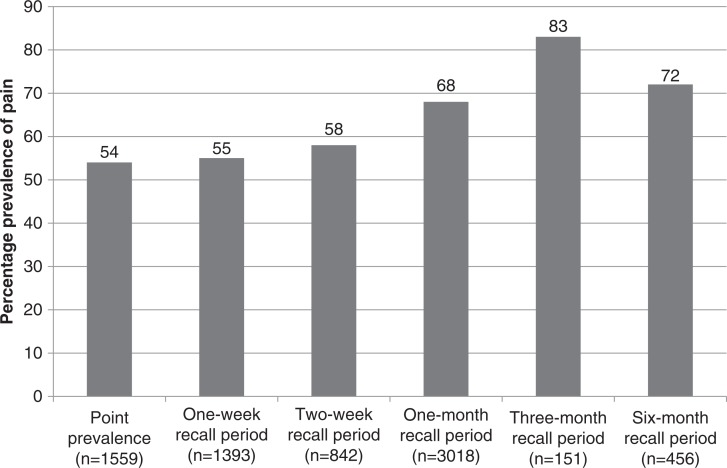
Prevalence of pain from pooled data.

The studies used a range of measurement instruments to record pain, with the well-validated Brief Pain Inventory (BPI) used in ten studies [13–16, 20, 30, 33, 34, 36, 38] and the comparable Wisconsin Brief Pain Questionnaire used in three studies [11, 26, 35]. The pain recall periods ranged from point prevalence to prevalence using a six-month recall period. One study did not report a recall period [17]. On analysis, there were no significant differences in prevalence of pain within each of the recall periods. The weighted prevalence of pain calculated from the pooled data ranged from 54% (95% CI 51.14–56.09) point prevalence of pain to prevalence rates of 55% (95% CI 57.71–62.84), 58% (95% CI 54.59–61.25), 68% (95% CI 65.9–69.24), 83% (95% CI 76–88) and 72% (95% CI 68.7–76.3) for one-week, two-week, one-month, three-month and six-month recall periods, respectively (Figure 3).

Of the 28 studies reviewed, 14 reported on anatomical sites of pain in 2077 participants. The number of anatomical sites reported ranged from a median of 1 (1–2) in the rural South African sample of the study by Mphahlele and colleagues [35] to a median of 2.5 (1–5) in the sample from New York reported on by Breitbart and colleagues [13, 14]. The relative frequencies of pain in different anatomical sites varied. All of the studies reporting sites of pain listed the lower limbs as a specific site of pain; the next most frequently reported site of pain was the head with 10 studies specifying headaches or head and neck pain. None of the studies reported on pain aetiologies.

Eighteen of the studies reported on severity of pain. In terms of pain characteristics, the pain reported by PLWHA is of moderate-to-severe intensity. Moderate-to-severe pain is recognized to have significant impact on ability to function and quality of life. Nine studies reported on pain interference with function [11–14, 20, 30, 35, 36, 38]. Pain interference scores from the BPI [no interference (0) – complete interference (10)] ranged from minimal interference in a group of urban South Africans [35] to moderate interference in a sample from the United States [13].

### Factors contributing to pain

Factors that contributed to the presence of pain in different samples were explored in several of the studies. Six studies reported on the contribution of demographic variables to the presence of pain [13, 24, 28, 31, 32, 34]. Two of the studies reported that women had a higher prevalence of pain than men [13, 34] with a further study identifying female intravenous drug users (IDUs) as having a higher prevalence of pain than male IDUs [24]. Conversely, Hitchcock and colleagues [28] found a higher prevalence of pain in males in their sample from Tshwane, South Africa. One study, reporting on female participants with CD4+<200, found no relationship between any demographic variables and pain prevalence [31].

With regard to ethnic groups, three studies with diverse ethnic groups reported a higher prevalence of pain in black participants [24, 32] and a further study reported a higher prevalence of pain in non-Caucasian groups [13]. Two studies found that lower levels of education increased pain prevalence [24, 34].

The relationship between stage of disease and the presence of pain was explored in eight of the studies reviewed [13, 17, 21, 24, 30–32, 38]. Two of the studies reported higher pain prevalence in participants with lower CD4+ counts on regression analysis [31, 32] and a third reported a correlation between number of pain sites and CD4+ count [17]. Three of the studies reported a higher prevalence of pain in participants with more advanced disease [Stage III or IV or Centre for Disease Control (CDC) category C] [17, 24, 30]. However, three other studies reported no differences between the prevalence of pain in different stages of the disease or according to CD4+ counts [13, 21, 38].

Two studies reported on the relationship between pharmacological treatment and the prevalence of pain [13, 31]. These three studies report conflicting results with Breitbart *et al*. [13] reporting that the ambulatory AIDS participants receiving ART in their study had a lower prevalence of pain than those not receiving ART. However, Richardson and colleagues’ [31] more recent work on women with AIDs found no difference in prevalence rates between those receiving and not receiving ART.

The relationship between a history of IDU and pain prevalence was reported in six studies [13, 17, 21, 24, 31, 33]. The earliest of these studies from Breitbart and colleagues [13] reported no differences in the prevalence of pain between participants with a history of IDU and non-IDU. However, the subsequent studies all report significantly higher rates of pain (prevalence and severity) in the IDU group [17, 21, 24, 31, 33]. In addition, sub-analysis in two studies revealed a clear relationship between the prevalence of pain and stage of disease in the non-IDU groups, which was not present in the IDU group [17, 21].

Five of the studies reviewed reported on psychological factors as contributors to pain [15, 19, 23, 31, 34]. All of these studies noted an association between the presence of psychological distress or illness and pain. Both greater levels of psychological distress and lower levels of emotional control were associated with greater levels of pain [15, 19, 23]. Two of the studies reported that pain severity was associated with worse depression scores [31, 34] and one noted that pain was associated with lower levels of perceived social support [15].

### Pain management

Six studies [14, 16, 18, 26, 35, 36] reported on adequacy of pain management using the PMI as described by Cleeland *et al*. [40] (Table 6). The PMI provides a score from −3 to +3 with scores ≥0 interpreted as adequate pharmacological management of pain. In all these studies, the majority of the participants were receiving inadequate pharmacological pain management (PMI<0) with results ranging from 100% of rural and urban South Africans with severe pain scoring PMI<0 [35] to 66% of HIV+ inpatients at a South African hospital scoring <0 [36].

**Table 6:** Summary of reported PMI scores

Author (year)references	Setting	Population (sample size)	Pain prevalence (%) (period)	PMI (%<0)
Larue *et al*. (1997) [16]	13 cities across France	HIV+ IP and OP (290)	62; 53; 30 (IP; DP; OP) (1 week)	85
Breitbart *et al*. (1997) [14]	New York, USA	Ambulatory AIDS patients (516)	63.6 (2 weeks) [67 (IDU); 59 (non-IDU)]	90.4 (IDU) 83.5 (non-IDU)
Frich & Borgbjerg (2000) [18]	Copenhagen, Denmark	AIDS patients at a Dept. of Infectious Diseases (95)	74 (point prevalence)	77
Aires & Bammann (2005) [26]	Sao Paulo, Brazil	HIV+ IP on admission (197)	54.3 (2 weeks)	83
Mphahlele *et al*. (2011) [35]	Urban and rural South Africa	HIV+ OP (rural 125; urban 396)	72 (rural) 66 (urban) (point prevalence)	100 (rural – severe pain) 100 (urban – severe pain) 100 (rural – moderate pain) 92 (urban – moderate pain)
Narasimooloo *et al*. (2011) [36]	Kwazulu-Natal South Africa	HIV+ IP urban district hospital (100)	91 (point prevalence)	66

IP=inpatient; OP=outpatient; DP=day patient; IDU=intravenous drug use; PMI=pain management index.

Three further studies reported on adequacy of pain management [11, 30, 38]. All three studies reported on the percentage of participants receiving no treatment for their pain. In a Canadian sample, 40% were receiving no analgesia for their pain [11], 60% of the sample from Nigeria were receiving no treatment [38] and 73% of the participants in the study from India were receiving no treatment for pain [30].

## Discussion

The main findings of this review were: (1) the prevalence of pain ranged from point prevalence of 54% (95% CI 51.14–56.09) to 83% (95% CI 76–88) using a three-month recall period; (2) the reported pain was of moderate-to-severe intensity, and pain was reported in one to two and a half different anatomical sites with moderate levels of pain interference with function; (3) diverse factors were found to contribute to pain; and (4) all nine studies reporting on the adequacy of pain management recorded marked under-treatment of pain.

There was a range in prevalence rates reported across the studies. The biggest variation was in point prevalence rates, which ranged from 10.9% of asymptomatic Vietnamese living with HIV reporting pain in a population survey [37] to 91% of HIV+ inpatients at an urban hospital in KwaZulu-Natal, South Africa, reporting pain [36]. Despite these ranges, on analysis, there were no differences in the median and mean prevalence rates of pain between the studies reporting point prevalence, or between the studies reporting one-week, two-week, one-month and three-month prevalence rates for pain. The consistency of the prevalence rates within each of the recall periods, from the earliest reviewed study published in 1993 [11] to the studies published in 2011, is also worth highlighting. Across all the prevalence periods (point prevalence through to one-month prevalence), prevalence rates have not decreased in recent years despite changes in pharmacological treatment of the disease.

Of the six studies reporting prevalence of pain using two-week recall periods, five used the BPI or the comparable Wisconsin instrument. These studies were conducted in diverse settings (low-and high-income countries) with samples of PLWHA ranging from asymptomatic HIV+ outpatients to inpatients with AIDS. The use of the same instrument facilitates pooling of the data and the mean prevalence rate of 58% for a two-week recall period may be a fair reflection of the prevalence for PLWHA in diverse settings at all stages of the disease.

The studies reviewed reported that PLWHA commonly present with multiple pain sites and a range of pain severity. Multiple pain sites suggest that there are several differing pathological processes contributing to pain at one time. This reinforces studies reporting various causes of pain in PLWHA, from the virus itself to opportunistic infections to side effects of treatment [2]. Although a growing body of research focuses on painful peripheral neuropathies in PLWHA [41], painful sites in the studies reviewed were not restricted to the peripheries, indicating that other mechanisms contributing to pain need to be explored.

The presence of severe pain in large portions of the samples in several studies is of concern because increased pain severity is associated with an increase in functional interference [28]. The functional impact of pain in PLWHA as reported in pain interference measures has consequences for health-related quality of life. Several studies used the pain interference score from the BPI to measure global pain interference. Sleep was the most commonly identified item, followed by interference with ability to work and interference with mood, emphasizing the global impact of pain on both the PLWHA and society. This underscores the need to address pain not simply to prevent suffering but to facilitate function.

Several factors were identified as contributing to pain. In studies reporting on populations from developed countries, a history of IDU, being female, and being of African-American descent increased risk of pain. Several studies reported that depression, anxiety, and a lack of social support increased risk of pain as did lower levels of education in populations from both developing and developed countries. The interplay of these variables suggests that the biopsychosocial model of pain is an appropriate paradigm from which to view pain in PLWHA.

Although the biopsychosocial model has been criticized for a lack of structure that would facilitate analysis of the weighted contribution of variables [42], in a complex construct such as pain in HIV/AIDS, it is still a useful conceptual framework from which to approach the problem, explore causes and establish effective treatment.

Despite an increasing awareness of pain as a problem for PLWHA, the problem of under-management persists. This raises at least two questions relating to the clinical management of PLWHA. The first is the question of whether the data on pain as a problem for PLWHA is being effectively distributed to clinicians working with this patient group. Alternatively, if the information is reaching the clinicians, under-treatment could result from a lack of effective pain management treatment strategies. Other barriers that may contribute to the under-management of pain in PLWHA are similar to those encountered in other conditions with pain, such as cancer, and include patients not realizing that the pain is related to their disease, fear of what the pain may mean, fear that the clinician may be distracted by the pain, fear of being labelled as a difficult patient and a lack of understanding that treatments other than highly active antiretroviral therapy are available [43, 44].

However, all responsibility does not lie with the patient; several clinician-related barriers have been identified, such as a lack of awareness about pain as a problem [18], a lack of access to adequate analgesia [45], fear of addictions [18, 46] and finally a lack of time in consultations [46]. Despite these barriers being identified, some of them as long as 10 years ago, data in the more recent studies reviewed indicate that pain management in PLWHA has not improved.

The increase in the number of studies published per year may be a reflection of a growing awareness of pain as a problem for PLWHA (Figure 2). There was not only an increase in quantity of papers but also in the methodological quality in recent years. This is perhaps not unexpected as the publication of guidelines for the reporting of observational studies (STROBE guidelines [6]) and similar papers have provided researchers with clear direction to assist in improving the quality of research.

Limitations of this review relate to the methods used to conduct the review and the quality of the data reviewed. The search terms used included the term “pain,” which may have introduced a selection bias into the search. Studies conducted on the impact of HIV/AIDS on functioning and participation, which could have explored pain as an impairment, may not have been identified using this search criteria. By using the term “pain” in the search, the search was biased towards finding studies reporting on the problem of pain. Studies that explored pain but did not identify it as a problem may not have used the term as a key word. The use of the term “impairment” in the search rather than “pain” may have limited this bias.

The search was restricted to articles published in English due to the language limitations of the researchers. This may have resulted in the exclusion of relevant literature from the review at the outset. Further, only one reviewer screened the full text articles and administered the 11-item methodological screening tool. Ideally, this procedure should be conducted by two independent researchers to ensure consensus in scoring and subsequent selection of studies. In the interests of reproducibility, only published studies were included in this review, and manual searching of journals not indexed in electronic databases was not performed. This could introduce a publication bias as unpublished studies and studies published in lower circulation journals may report results against the trend of those previously published.

One component not explored in the studies reviewed was the non-pharmacological management of pain. Pain management interventions using exercise [47–50], relaxation or mindfulness-based techniques, cognitive behavioural therapy and patient education have been recommended for PLWHA [51–54].

## Conclusions

The aims of this systematic review were to establish the prevalence of pain, characteristics and contributing factors to pain, whether pain is adequately treated and the functional impact of pain in PLWHA. The results of this review highlight that pain is present among 55%– 67% of PLWHA at all stages of the disease, with prevalence rates increasing with the length of the recall period. The prevalence rates for pain in PLWHA do not appear to have diminished over the 30 years spanning the studies reviewed despite large scale roll-out of ART world-wide.

This review highlighted several factors researchers should consider in planning further research into pain in PLWHA. The under-representation of populations from low-income countries and females in the studies reviewed indicates the need for further work in these groups, with specific attention to ethnicity and cultural factors. Further detail regarding pain in HIV/AIDS is required, particularly an exploration of the sites, types and aetiological contributors to pain.

It is a matter of concern that despite the institution of an international Task Force on Pain and AIDS in 1994 [5], there is still marked under-treatment of pain in PLWHA. The dissemination of the systematic presentation of cumulative data in this review might be useful to motivate clinicians to address this symptom and its effects in PLWHA by emphasizing the severity and scale of the problem of pain in PLWHA.

The body of work available in the literature thus far, while emphasizing the problem of pain for PLWHA does not appear to have had an impact on the management of this symptom. The lack of association between pain and pharmacological treatment of HIV (ART) indicates that treatment or management of the virus is not sufficient to manage pain. Thus specific assessment of pain at each clinical visit is recommended, along with clear guidelines for the pharmacological and non-pharmacological management of any pain present.
